# Immobilized Trienzymatic System with Enhanced Stabilization for the Biotransformation of Lactose

**DOI:** 10.3390/molecules22020284

**Published:** 2017-02-22

**Authors:** Pedro Torres, Francisco Batista-Viera

**Affiliations:** Cátedra de Bioquímica, Departamento de Biociencias, Facultad de Química, Universidad de la República, Gral Flores 2124, 11800 Montevideo, Uruguay; ptorres@fq.edu.uy

**Keywords:** β-galactosidase, l-arabinose isomerase, d-glucose isomerase, enzyme immobilization, d-tagatose, d-fructose

## Abstract

The use of ketohexose isomerases is a powerful tool in lactose whey processing, but these enzymes can be very sensitive and expensive. Development of immobilized/stabilized biocatalysts could be a further option to improve the process. In this work, β-galactosidase from *Bacillus circulans*, l-arabinose (d-galactose) isomerase from *Enterococcus faecium*, and d-xylose (d-glucose) isomerase from *Streptomyces rubiginosus* were immobilized individually onto Eupergit C and Eupergit C 250 L. Immobilized activity yields were over 90% in all cases. With the purpose of increasing thermostability of derivatives, two post-immobilization treatments were performed: alkaline incubation to favor the formation of additional covalent linkages, and blocking of excess oxirane groups by reacting with glycine. The greatest thermostability was achieved when alkaline incubation was carried out for 24 h, producing l-arabinose isomerase-Eupergit C derivatives with a half-life of 379 h and d-xylose isomerase-Eupergit C derivatives with a half-life of 554 h at 50 °C. Preliminary assays using immobilized and stabilized biocatalysts sequentially to biotransform lactose at pH 7.0 and 50 °C demonstrated improved performances as compared with soluble enzymes. Further improvements in ketohexose productivities were achieved when the three single-immobilizates were incubated simultaneously with lactose in a mono-reactor system.

## 1. Introduction

Biological processes offer multiple advantages compared with conventional chemistry [1]. Proper and well-designed enzyme immobilization processes can improve the characteristics, capacity, and performance of biocatalysts in aspects such as reuse, stability, prevention of product contamination with enzyme, minimization of allergenicity, generation of new catalytic properties, increased specificity and selectivity, among others [2,3,4,5]. Immobilization strategies include molecular cross-linking, encapsulation, entrapment, and binding to solid supports by physical adsorption or formation of covalent linkages [2,5].

Commercial epoxy-activated acrylic resins such as Eupergit^®^ C and Eupergit^®^ C 250 L have excellent properties for covalent immobilization of proteins, and in some cases provide good stabilization of the biomolecules. The above-mentioned supports are formed by spherical macroporous particles possessing large internal surfaces and characterized by a low water uptake. They differ in their internal morphology and epoxy group content, which determine important differences in their properties. Eupergit C 250 L has larger pores and a lower number of oxirane groups than Eupergit C [6,7].

Protein immobilization onto epoxy-activated acrylic supports occurs as a two-step mechanism. The primary event is rapid physical adsorption of the protein onto the resin; in a second step, a covalent reaction takes place between the adsorbed protein and the support [8,9]. Epoxy groups react with amino groups in proteins under very mild experimental conditions (e.g., pH 7.5), with minimal chemical modification of the protein and formation of very stable secondary amine bonds [6,7,8,9]. Multipoint covalent attachment followed by blocking the remaining epoxide moieties with hydrophilic reagents can generate even more stable enzyme derivatives under controlled conditions [3,8,9]. The geometric characteristics of epoxy activated resins also favor multisubunit immobilization [3].

Many efforts have been focused on the development and application of covalent immobilization technologies for the biotransformation of lactose, the main carbohydrate of milk, whey, and dairy products. Lactose is a disaccharide of very low solubility and low sweetness compared with its hydrolysis products, d-glucose and d-galactose. In the food industry, lactose hydrolysis increases solubility and sweetness, resulting in improved sensory characteristics of foods containing hydrolyzed lactose from milk or whey. A major consideration is that more than 70% of human adults worldwide have intestinal dysfunction when they consume milk [10].

The enzymatic hydrolysis of lactose by β-galactosidases (EC 3.2.1.23) takes place in mild conditions and does not cause undesired flavors, odors, or colors. However, enzyme inhibition by the reaction products remains one of the main disadvantages of reactions catalyzed by β-galactosidases. Current research into the processing of dairy products is focused on the biotransformation of lactose into products of interest other than glucose and galactose [11].

Commercial preparation of β-galactosidase from *Bacillus circulans* (Biolacta N-5, Daiwa Kasei, Osaka, Japan) consists of various isoforms, but β-galactosidase-I is the main form in terms of abundance [12]. The activity of this isozyme is not affected by an ionic environment (an advantage compared with certain *Kluyveromyces* β-galactosidases that are inhibited by divalent calcium); its optimal reaction conditions are 44 °C and pH 5.5–6.5 [13]. The enzyme also catalyzes the regioselective synthesis of oligosaccharides by formation of β (1–4) linkages [14,15]. Recently, the β-galactosidase from *Bacillus circulans* was covalently immobilized onto a commercially available matrix, Eupergit C 250 L, with high protein immobilization yields (90%–99%) and high activity immobilization yields (around 80%–90%) [16].

l-Arabinose isomerase (EC 5.3.1.4) is an intracellular enzyme that catalyzes the partial isomerization of l-arabinose to l-ribulose in vivo [17,18,19,20,21,22]. In vitro experiments have shown that this biocatalyst can also convert d-galactose to d-tagatose, a rare sugar with promising nutraceutical properties [23,24]. d-Tagatose is a ketohexose of great interest for both technological and nutritional reasons, and many alternative processes for obtaining it, apart from chemical synthesis [25], have been studied in recent years [23]. Biological conversion of d-galactose to d-tagatose employing the enzyme l-arabinose isomerase is the most economically viable biological d-tagatose manufacturing process so far. However, biological production of d-tagatose requires the development of optimized biotransformation processes with stabilized biocatalysts [26,27]. It has recently been reported that l-arabinose isomerase from *Enterococcus faecium,* a bacterial enzyme with interesting properties, was produced and purified by affinity techniques [28,29].

d-Xylose (d-glucose) isomerase (d-xylose keto isomerase, EC 5.3.1.5) is an intracellular enzyme that catalyzes the partial and reversible in vivo isomerization of d-xylose into d-xylulose and in vitro of d-glucose into d-fructose [22], and therefore has the potential to transform glucose, the other product (along with galactose) of enzymatic lactose hydrolysis. 

Certain authors have explored the potential of the combined use of these biocatalysts; e.g., Rhimi et al. (2007) reported the co-expression in *E. coli* of l-arabinose isomerase from *Bacillus stearothermophilus* US100 and a mutant d-xylose isomerase from *Streptomyces* SK followed by cell immobilization on alginate beads [22]; and Xu et al. (2016) developed the co-expression of the β-galactosidase from *Thermus thermophilus* HB27 and the l-arabinose isomerase from *Lactobacillus fermentum* CGMCC 2921 [27].

The efficient immobilization or co-immobilization of enzymes that participate in cascade reactions can mimic or conveniently modify in vitro processes from a technological point of view [30]. However, the choice of a particular strategy strongly depends on each case, even for one pot configuration. While co-immobilization may be preferred because of the local increase in concentration of secondary substrates and derived kinetic advantages, the intrinsic complexity of the system—including differences in multiple enzyme stability, requirements for homogeneous immobilization protocols, and reduction in stability due to undesired interactions—creates great difficulties for some applications [31,32]. 

In the present paper, we report the development of a tri-enzymatic system composed of β-galactosidase, d-xylose (d-glucose) isomerase, and l-arabinose (d-galactose) isomerase, immobilized individually onto oxirane-carrying Eupergit resins [6]. The resulting complex biocatalysts were characterized. Functional characteristics were determined and compared to those for soluble enzymes for each of the enzymes separately immobilized by covalent attachment to Eupergit C or Eupergit C 250 L. Subsequently, the capacity of the single-immobilizates to produce d-tagatose and d-fructose from whey lactose under sequential or mono-reactor operative conditions was studied. 

## 2. Results and Discussion

**Figure 1:**
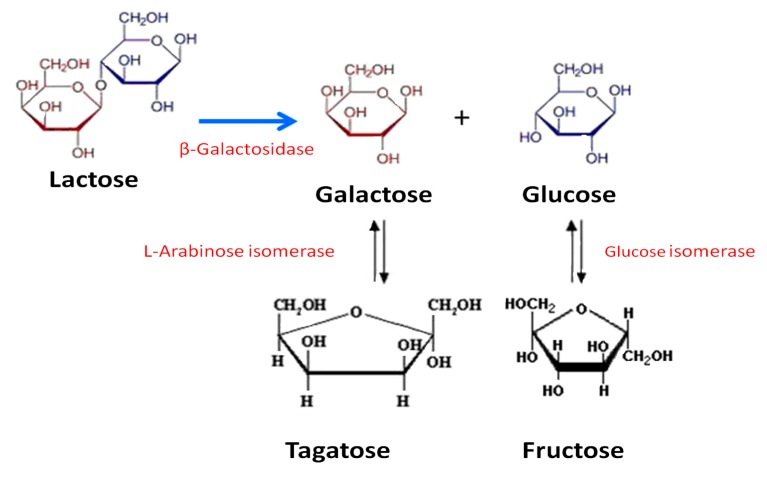
Diagram of reactions for enzymatic production of ketohexoses from lactose. Structures of main products of interest are depicted with enlarged sizes.

In this work, we have explored the combined use of β-galactosidase biocatalysts developed previously [16], with immobilized d-xylose (d-glucose) and l-arabinose (d-galactose) isomerases to achieve the partial biotransformation of lactose into the ketohexoses d-fructose and d-tagatose (Figure 1).

### 2.1. Immobilization and Stabilization

#### 2.1.1. l-Arabinose Isomerase

Purified l-arabinose (d-galactose) isomerase was immobilized onto acrylic epoxy-activated supports (Eupergit C and Eupergit C 250 L) by a similar strategy to that used for the immobilization of β-galactosidase [16], achieving maximum immobilization yields (100%) and high activity immobilization yields (around 90%). 

The influence of blocking remaining epoxy groups in derivatives with glycine, or alkaline treatment at pH 8.5 and subsequent blocking on the activity (in terms of immobilized activity yield) and stability (in terms of half-lives at 50 °C) was studied (Table 1).

Blocked derivatives retained around 70% (Eupergit C) and almost 90% (Eupergit C 250 L) of immobilized activity, and the stabilities of these derivatives were clearly higher than those corresponding to native enzymes and the unblocked derivatives (Table 1).

A substantial reduction in immobilized activity yield (e.g., from 89% to 73% for Eupergit C 250 L derivative) was observed after alkaline incubation compared with control blocked derivatives without alkaline treatment, as has already been reported [33,34]. Nevertheless, the stability of derivatives clearly improved after alkaline treatment and blocking. The best thermostability was achieved when alkaline incubation was carried out for 24–28 h before blocking (half-lives at 50 °C of 308 and 379 h for Eupergit C 250 L and Eupergit C derivatives respectively, as compared with 65 and 95 h for the corresponding blocked derivatives). The substantial reduction in the expressed activity after stabilization by the combined strategy was compensated for by the significant gain in stability. These stabilized derivatives could work at moderately high temperatures, favoring tagatose production.

#### 2.1.2. d-Xylose Isomerase

d-Xylose (d-glucose) isomerase from Streptomyces rubiginosus (Hampton) was immobilized onto Eupergit C and Eupergit C 250 L by the same strategy described for the other biocatalysts, achieving maximum immobilization yields (100%) and immobilized activity yields (91% for Eupergit C and 98% for Eupergit C 250 L). Thermal stabilization of these biocatalysts was performed by post-immobilization strategies as described previously for l-arabinose isomerase (Table 2). 

Eupergit C 250 L and Eupergit C blocked derivatives retained 98% and 91% of their original immobilized activity, respectively. Half-lives at 50 °C were 210, 230, and 23 h for Eupergit C 250 L, Eupergit C derivatives and free enzyme, respectively, exhibiting moderate (approximately 10-fold) stabilization of immobilizates. Greater stabilization was achieved in the case of derivatives with 24 h alkaline incubation before blocking (half-lives of 491 and 554 h at 50 °C for Eupergit C 250 L and Eupergit C derivatives, respectively). Immobilized activity yields decreased for these derivatives, especially in the case of Eupergit C (a more activated support), reinforcing the hypothesis that intense multipoint covalent attachment was responsible for differences in stability. In addition, the homotetrameric structure of d-xylose (d-glucose) isomerase from *S. rubiginosus* [35] could explain its greater stabilization levels with respect to monomeric β-galactosidase, as hypothesized also in the case of tetrameric l-arabinose isomerase. 

### 2.2. Characterization of Derivatives

#### 2.2.1. Optimum pH and pH Stability Profile

The influence of pH on the activity and stability of the immobilized, stabilized and blocked derivatives was analyzed in comparison with soluble enzymes. Derivatives used for this purpose were prepared under the conditions described in the Materials and Methods section.

**Figure 2:**
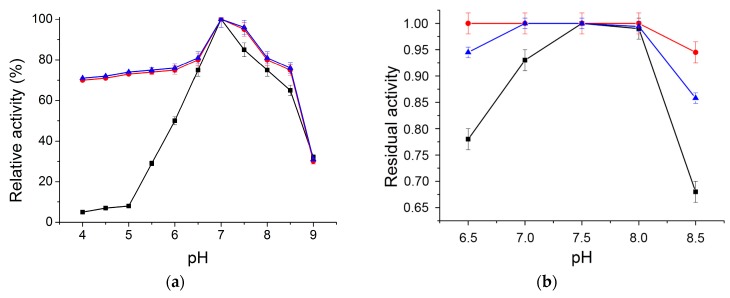
Influence of pH on (**a**) activity and (**b**) stability of free l-arabinose (d-galactose) isomerase (LAI) and insoluble derivatives. ■ native enzyme; ▲ LAI-Eupergit C derivative; ● LAI-Eupergit C 250 L derivative.

Free l-arabinose isomerase and d-xylose isomerase showed optimum pH of 7.0 and 7.5, respectively, as previously reported [28,36]. Activity of soluble d-xylose isomerase decreased at acidic pH. 

d-Xylose isomerase derivatives onto Eupergit C and Eupergit C 250 L showed a similar profile, with maximal activity at pH 7.5, and higher activity than the soluble form at acidic pH (below 6.0). l-Arabinose derivatives onto Eupergit C and Eupergit C 250 L exhibited maximal activity in the pH range 7.0–7.5, with higher activity than soluble enzyme at pH below 6.5 (Figure 2a). This behavior could be due to the influence of the new microenvironment, although it is probably also highly dependent on the nature of the enzyme. For example, immobilization of β-galactosidase from *B. circulans* onto Eupergit supports produced a shift in optimum pH [37], but immobilized trehalose synthase exhibited no change in the pH of maximal activity [38].

The stability profiles of free l-arabinose isomerase and insoluble derivatives immobilized on Eupergit C and Eupergit C 250 L showed a maximum in the pH range 7.0–7.5 (Figure 2b). Derivatives exhibited higher stability than soluble enzyme in the range 6.5–8.5. Furthermore, stabilization by alkaline incubation and blocking was also effective for this wide pH range. In all cases, Eupergit C 250 L derivatives displayed better stabilities than Eupergit C derivatives. On the other hand, d-xylose isomerase derivatives exhibited only slight pH stabilization and alkaline incubation had no effect on pH stability profiles. 

Concerning stability, immobilized d-xylose isomerase retained more than 75% of activity when incubated 8 h at pH in the range 5.0–8.5. Native enzyme remained only about 40% active under the same conditions.

#### 2.2.2. Optimum Temperature and Thermal Stability

As reported previously, optimum temperature for free l-arabinose isomerase was 50 °C [28]. Derivatives exhibited an optimum temperature around 53 °C, and maintained activities of more than 90% of maximum at temperatures up to 55 °C.

**Figure 3:**
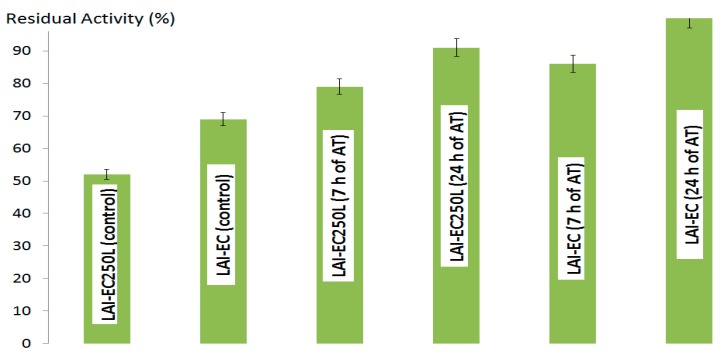
Thermal stability of l-arabinose (d-galactose) isomerase (LAI) derivatives onto Eupergit C (EC) and Eupergit C 250 L (EC250L) with alkaline treatment (AT) and blocking with glycine. Derivatives were incubated for 30 h at 60 °C and residual activities were determined. Control derivatives were only blocked but did not receive alkaline treatment after immobilization.

The free enzyme l-arabinose isomerase lost between 82% and 94% of its initial activity after 6 h incubation at 50 °C and 60 °C, respectively. Thermostabilities of glycine-blocked derivatives were clearly higher than those corresponding to native enzyme and unblocked derivatives. The combination of post-immobilization alkaline incubation at pH 8.5 for 7–28 h and blocking increased thermal stability. Figure 3 shows the residual activities of stabilized derivatives after 30 h incubation at 60 °C. 

The length of post-immobilization alkaline treatment determined the increase in stability. Stability was also influenced by the support, i.e., derivatives onto Eupergit C were more stable than Eupergit C 250 L derivatives. However, these highly stabilized derivatives exhibited a consistent reduction in immobilized activity yield. These facts are consistent with the hypothesis that intense multipoint attachment occurred between enzyme and support during alkaline incubation under the conditions described, which produced a certain degree of inactivation and resulted in structural rigidification, providing increased thermal stability [8,9]. 

Thermal stabilization was higher than that obtained for β-galactosidase from *B. circulans* [16], probably due to the multimeric nature of l-arabinose isomerase. Stabilization in this case depended on rigidification, multisubunit immobilization, and hydrophilization of microenvironment, phenomena which have been extensively reported [8,9,38,39,40,41]. 

In the case of d-xylose isomerase, free enzyme and derivatives exhibited a continuous rise in activity in the range 40–70 °C. Optimum temperature for d-xylose (d-glucose) isomerase immobilized onto Eupergit C and Eupergit C 250 L was 60 °C, as reported previously [36]. Despite the high thermal stability of d-xylose (d-glucose) isomerase, the enzyme was further stabilized 30- to 40-fold based on the strategies described in this study, combining immobilization, post-immobilization alkaline treatment, and blocking (Table 2).

#### 2.2.3. Kinetic Parameters

K_m_ for free enzymes and apparent K_m_ for Eupergit C and Eupergit C 250 L derivatives were determined at 30 °C, 40 °C, and 50 °C, using d-galactose (l-arabinose isomerase) or d-glucose (d-xylose isomerase) as substrates. Michaelis-Menten kinetics were assumed and parameters were calculated after Eadie-Hofstee linearization. Apparent K_m_ values for derivatives were higher than the K_m_ for free enzyme, and both decreased when temperature increased (Table 3 and Table 4).

In the case of l-arabinose isomerase, Eupergit C derivatives showed higher K_m_ and lower V_max_ than Eupergit C 250 L derivatives (Table 3). Greater pore size in Eupergit C 250 L [6,7] may favor the access of substrate to the active site, especially in the case of enzyme molecules immobilized onto internal surfaces. The K_m_ value at 50 °C (34 mM for free enzyme and 40 mM for Eupergit C 250 L derivative) was lower than K_m_ for most l-arabinose isomerases reported to date (55 to 400 mM). 

Free enzyme and insoluble derivatives were not inhibited by d-tagatose (0–500 mM), representing a technological advantage.

In the case of d-xylose isomerase, Eupergit C derivatives also exhibited lower V_max_ than Eupergit C 250 L derivatives (Table 4), which could be explained by diffusional or steric restrictions, structural modification of enzyme by the immobilization procedure, or low accessibility of substrate to the active site, as discussed above for l-arabinose isomerase. 

### 2.3. Performance of Biocatalysts

#### 2.3.1. d-Galactose and d-Glucose Isomerization by Immobilized Ketohexose Isomerases

**Figure 4:**
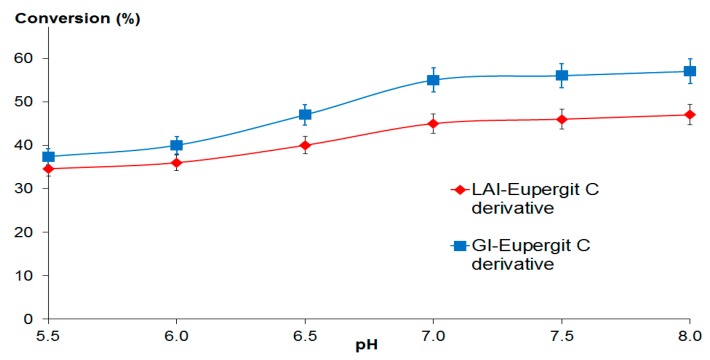
Influence of pH on the activity of stabilized l-arabinose (d-galactose) isomerase (LAI) and d-xylose (d-glucose) isomerase (GI) immobilized onto Eupergit C during batchwise operation at 60 °C with d-galactose and d-glucose (100 g/L), respectively.

Eupergit C and Eupergit C 250 L derivatives of l-arabinose isomerase and d-xylose isomerase were used for batch isomerization of d-galactose or d-glucose (100 g/L), respectively, in 0.1 M phosphate buffer at temperatures in the range 30–60 °C and pH from 5.5 to 8.0. For l-arabinose isomerase derivatives, conversion percentages achieved at pH 7.0 were 26% at 30 °C and 45% at 60 °C. In the case of d-xylose isomerase derivatives, conversion percentages at neutral pH were higher, attaining 36% of conversion at 30 °C and 55% at 60 °C, respectively, similar to results reported in earlier studies [36]. Figure 4 shows the influence of operational pH on the degree of conversion obtained at 60 °C for l-arabinose and d-xylose isomerases immobilized onto Eupergit C. Conversions in the pH range studied (5.5–8.0) were above 35% in all cases, with best performances at pH 7.0–8.0.

#### 2.3.2. Lactose Bioconversion

Sequential application of single-immobilizates of β-galactosidase, l-arabinose isomerase, and d-xylose isomerase derivatives to biotransform 4.6% lactose in phosphate buffer pH 7.0, in separate bio-reactors, resulted in conversion percentages in terms of d-tagatose and d-fructose after the first 6 h operation at 50 °C (determined by HPLC analysis) of 31% and 24%, respectively (Table 5). 

Ketohexose production was also studied using soluble enzymes sequentially under similar conditions. For soluble enzymes, the kinetics were faster, but productivity and conversion degree were lower than for the immobilized biocatalysts, suggesting that stabilization by immobilization also plays an important role in this case. 

**Figure 5:**
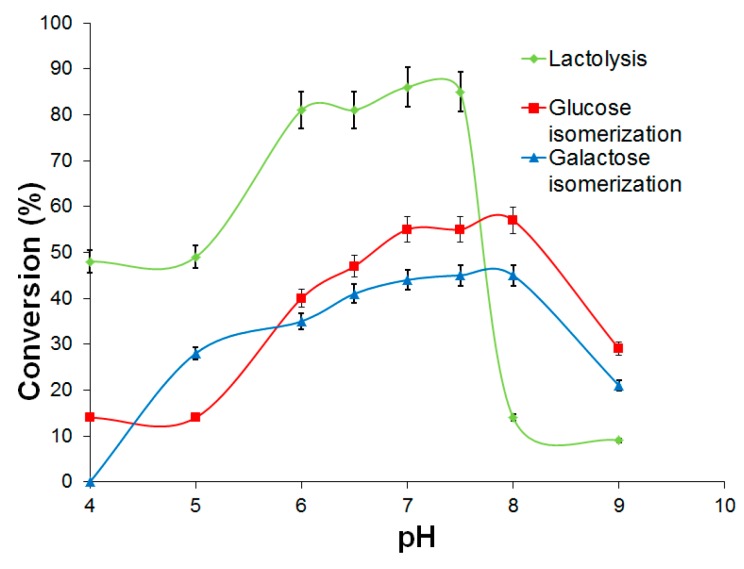
Lactolysis, glucose isomerization and galactose isomerization achieved by using the biocatalysts β-galactosidase, l-arabinose (d-galactose) isomerase (LAI), and d-xylose (d-glucose) isomerase (GI) immobilized onto Eupergit C (EC) sequentially at pH 4–9, during batchwise operation at 60 °C with 4.6% lactose as the starting substrate.

The influence of pH was studied by performing the lactose hydrolysis and isomerization processes at different pHs in the range 4–9 (Figure 5) and analyzing the results in terms of degree of lactolysis and the corresponding percentages of d-fructose and d-tagatose produced under these conditions at 60 °C.

The best performance of β-galactosidase derivatives occurred between pH 6.0 and 7.5, which were also favorable conditions for isomerization reactions (Figure 5), but lactose hydrolysis was incomplete in all experiments employing these biocatalysts sequentially, because of product inhibition due to galactose and glucose. 

A natural consequence of the increase in operational temperature is to achieve better conversion degrees. However, the percentages obtained at pH 7.0 and 50 °C (conditions that favored the operational stability of derivatives) remained lower than 50% and 40% for fructose and tagatose, respectively. Multiple strategies to solve product inhibition via immobilization were assayed, involving rigidification of enzyme structure, modification of microenvironments, distortion/blocking of inhibition sites, use of thermophilic enzymes, and removal of inhibitors, among others [31,40,41,42,43], and the last-mentioned was selected in this work.

The productivity of primary lactolysis affected the productivity of subsequent steps, but we hypothesized that simultaneous lactolysis and isomerization by single-immobilizates or co-immobilizates could reduce inhibition to achieve higher conversion rates and higher productivity. With the purpose of preliminary confirmation of this fact, β-galactosidase, l-arabinose (d-galactose) isomerase, and d-xylose (d-glucose) isomerase immobilized individually onto Eupergit C and Eupergit C 250 L were used batchwise in mono-reactor systems for lactolysis/isomerization. The degrees of lactolysis achieved with buffered lactose or whey at 50 °C were significantly higher than those obtained by using a mono-enzymatic process with immobilized β-galactosidase (i.e., 93% and 86%, respectively, with lactose from Mozzarella whey as substrate). Hence, the conversion levels in mono-reactor systems were significantly higher than in multi-reactor ones, both in terms of lactose hydrolyzed and total ketohexoses formed (Table 5). 

Final productivities in terms of tagatose were 9.1 g/Lh for mono-reactor systems based on single-immobilizates. These productivity values were comparable with that obtained by co-expression in *E. coli* of the β-galactosidase from *Thermus thermophilus* HB27 and the l-arabinose isomerase from *Lactobacillus fermentum* CGMCC 2921, which yielded 6.3 g/Lh in the presence of borate [27]. Co-expression in the same host of thermostable l-arabinose isomerase of *Bacillus stearothermophilus* US100 and a mutant d-xylose isomerase of *Streptomyces* SK followed by cell entrapment on alginate beads achieved 3.0 g/Lh of ketohexoses as optimal productivity at 65 °C and pH 7.5 [22].

The tri-enzymatic mono-reactor system operating with soluble enzymes maintained higher rates than single-immobilizate mono-reactor systems, but productivity and conversion percentages for lactolysis (76%) or isomerization (22% and 21% for tagatose and fructose after the first 6 h) were reduced, with d-tagatose production at the lowest level (Table 5). These results were determined mainly by the poor thermal stability of soluble l-arabinose isomerase. The reduced stability of this enzyme also determines the observed decrease in lactolysis percentages (due to accumulation of galactose, a potent inhibitor of β-galactosidase in the medium under these conditions). On the other hand, fructose production was less influenced, but d-glucose has less influenced as an inhibitor of β-galactosidase than d-galactose [43].

In fact, immobilized β-galactosidase and d-xylose isomerase derivatives were more stable (and so less expensive) than l-arabinose isomerase. The degree of lactolysis over repeated cycles of use becomes an important index, providing information on the deactivation of the d-galactose-consuming enzyme, and even determining the addition of individual fresh derivative. For a covalent immobilization strategy as described, the work with co-immobilizates could represent an advantage in several ways [30,44], but the inactivation of the most labile enzyme will determine the operative half-life of the whole biocatalyst and the discarding of the other enzymes while they are still active [31].

In a forthcoming paper, we will further refine these studies by analyzing and comparing various alternative systems for achieving lactose biotransformation, working either with buffered lactose or whey as substrates.

## 3. Materials and Methods

### 3.1. Materials

The enzyme β-galactosidase from *B. circulans* (Biolacta N-5) was kindly provided by Daiwa Kasei (Osaka, Japan), d-xylose (d-glucose) isomerase was purchased from Hampton Research (Aliso Viejo, CA, USA). Eupergit C and Eupergit C 250 L were a gift from Röhm Pharma (Darmstadt, Germany). *O*-Nitrophenyl-β-d-galactopyranoside (ONPG), α-lactose, d-galactose, d-tagatose, d-glucose, d-fructose, l-cysteine hydrochloride, and carbazole were purchased from Sigma (St. Louis, MO, USA); bicinchoninic acid (BCA reagent) was from Pierce (Rockford, IL, USA); and all other chemicals were of analytical or HPLC grade. The enzymatic kit for glucose determination was from Spinreact S.A. (Girona, Spain). Cheese wheys were kindly supplied by CONAPROLE (Cooperativa Nacional de Productores de Leche, Uruguay).

### 3.2. Sugar Analysis

The glucose and the total ketohexose contents in the samples were measured by Trinder’s assay and the cysteine-carbazole-sulfuric acid method, respectively [45,46]. 

Analysis and characterization of sugars by HPLC was performed in a Waters-Millipore apparatus (Waters-Millipore Corp., Burlington, NC, USA) equipped with a carbohydrate analysis column (Rezex RCM, Phenomenex, Torrance, CA, USA) and a RI-detector, at 80 °C and a flow rate of 0.6 mL/min, employing distilled water as mobile phase and lactose, d-glucose, d-galactose, d-tagatose, and d-fructose as standards (Sigma Chem. Co., St. Louis, MO, USA).

### 3.3. Protein Determination

The protein concentration in soluble samples and gel derivatives were determined using the BCA reagent [47,48,49]. 

### 3.4. Soluble Enzyme Treatment

The commercial β-galactosidase was partially purified as indicated by Torres and Batista-Viera [16]. The crude extract of l-arabinose isomerase from *E. faecium* was obtained as described in Manzo et al. [28] and purified by affinity chromatography on l-arabitol-agarose [29]. The commercial enzyme preparation of d-xylose isomerase was diluted 10-fold in 0.1 M potassium phosphate buffer pH 7.5, and gel filtered on Sephadex G-25 to remove ammonium sulphate and low molecular weight additives.

### 3.5. Enzyme Immobilization

Immobilization of each one of the three enzymes onto Eupergit C or Eupergit C 250 L was carried out at 25 °C with shaking in 1 M potassium phosphate buffer pH 7.5 for 24 h. Applied loads were 30 mg/g of gel (β-galactosidase), 4 mg/g of gel (l-arabinose isomerase), and 2 mg/g of gel (d-xylose isomerase). Derivatives were collected on a sintered-glass filter and washed with 1 M potassium phosphate buffer pH 7.5 and 0.1 M sodium phosphate buffer pH 7.5. The enzyme derivatives were stabilized by alkaline treatment and blocking with glycine as indicated in a previous report [16]. 

“Protein Immobilization Yield” was defined as the ratio (as percentage) of the amount of protein found on the gel to the amount of protein applied. “Immobilized Activity Yield” was defined as the ratio (as percentage) of the activity expressed by the gel derivative to the amount of total applied activity. Thus, “Protein Immobilization Yield” and “Immobilized Activity Yield” for the three enzymes were calculated as [P_gel_/P_applied_] × 100 and [A_gel_/A_applied_] × 100, where P corresponds to protein amount and A to enzyme activity [16]. 

### 3.6. Enzyme Assays

β-Galactosidase and lactase activities as well as l-arabinose (d-galactose) isomerase activity for soluble or immobilized enzymes were assayed as previously reported [16,28,29]. d-Glucose isomerase activity was determined by measuring the amount of d-fructose generated from d-glucose. The reaction mixture contained 5 mM MgCl_2_, 1 mM CoCl_2_, 500 mM d-glucose, 200 µL of appropriately diluted enzyme preparation, and 50 mM sodium phosphate buffer pH 7.5 to bring the final volume to 1 mL. The assay was done by incubating the reaction mixture at 25 °C for 1 h [42]. The enzymatic reaction was stopped (in the case of soluble enzyme) by boiling the samples for 3 min. The amount of d-fructose produced was determined spectrophotometrically at 560 nm by the cysteine-carbazole-sulfuric acid method [46]. The immobilized enzyme activity was assayed by the same method. One unit of glucose isomerase activity was defined as the amount of enzyme catalyzing the formation of 1 µmol of keto-sugar per minute under the specified conditions.

### 3.7. Properties of Insoluble Derivatives

The influence of pH and temperature on the activity and stability of native enzymes and immobilized derivatives was studied. 

#### 3.7.1. Effect of Temperature on Activity

The influence of temperature on the activity of soluble enzymes and immobilized derivatives was determined in the range of 20–60 °C under the pH conditions applicable for each assay. For assays, amounts of 20–50 mg of insoluble filter dried biocatalyst were used.

#### 3.7.2. Thermal Stability

The thermostability assays were performed at different temperatures in the range 20–60 °C in activity buffer, by incubating soluble enzymes or immobilized derivatives in a shaking bath. The inactivation kinetics of biocatalysts were evaluated for 1 to 55 h, and the corresponding half-lives calculated, according to the Sadana-Henley model [50], as described in a previous report [16]. 

#### 3.7.3. Optimum pH and pH Stability

The influence of pH on the activity of native enzymes and insoluble derivatives was monitored in the pH range 4–9 at 25 °C, using the corresponding substrate, according to previous reports [16,28,29]. The effect of pH on stability was determined in the same pH range by measuring the residual activity after incubating free or immobilized catalyst at the selected pH for 1–24 h at 25 °C. For pH activity or stability assays of insoluble derivatives, amounts of 20–50 mg of filter dried biocatalyst were used. In addition, the effect of pH was evaluated under operative conditions at 50–60 °C in the range 4.0–9.0.

#### 3.7.4. Determination of Kinetic Parameters

The kinetic parameters were determined using the corresponding substrate (0–500 mM) in 0.1 M phosphate buffer pH 7.0 at temperatures in the range of 30–50 °C, and calculated according to the Michaelis-Menten model, applying the Eadie-Hofstee linearization. Possible product inhibition of l-arabinose isomerase by d-tagatose was tested for initial concentrations of 0–500 mM.

### 3.8. Applications with Substrates

The native enzymes and the immobilized derivatives were used in three-step or one-step batch processes with the following substrates in 0.1 M phosphate buffer: d-glucose (100 g/L), d-galactose (100 g/L), lactose (46 g/L, equivalent to the concentration of lactose generally available in non-fermented fluid dairy products), and Mozzarella cheese whey. In the sequential three-step application, after lactose was hydrolyzed by β-galactosidase (step 1), the hydrolysis products were partially isomerized to d-tagatose and d-fructose by l-arabinose isomerase (step 2) and d-xylose isomerase (step 3). In the simultaneous processes, the three activities operated in a single bio-reactor. The activity of the immobilized enzyme derivatives was determined under different conditions of pH and temperature, and compared with the native enzymes. 

## 4. Conclusions

In conclusion, β-galactosidase from *B. circulans*, l-arabinose isomerase from *E. faecium*, and d-xylose isomerase from *S. rubiginosus* were immobilized individually onto Eupergit C and Eupergit C 250 L with high immobilization yields (around 100%) and high immobilized activity yields (around 80%–90%). Decrease in expressed activity after stabilization by a combined strategy (alkaline incubation for 24 h followed by blocking with glycine) was compensated for by the significant gain in thermal stability. This effect was most important in the case of the very sensitive l-arabinose isomerase, with half-lives at 50 °C of 308 and 379 h for Eupergit C 250 L and Eupergit C derivatives, respectively. These stabilized derivatives could work at moderately high temperatures, favoring tagatose production. The homotetrameric structure of d-xylose (d-glucose) isomerase and l-arabinose (d-galactose) isomerase could explain their higher stabilization levels with respect to monomeric β-galactosidase. 

Sequential application of single-immobilizates of β-galactosidase, l-arabinose isomerase, and d-xylose isomerase to biotransform lactose at pH 7.0 achieve conversion percentages in terms of d-tagatose and d-fructose of 31% and 24%, respectively after 6 h operation at 50 °C. Soluble enzymes under a similar operation mode had lower productivity and conversion degrees than using the immobilized biocatalysts, showing better performance of immobilizates. However, the sequential use of the biocatalysts resulted in incomplete lactose hydrolysis because of product inhibition by d-galactose and d-glucose. On the other hand, a mono-reactor system with a combination of the three derivatives favored higher conversion percentages, especially for the lactolysis reaction, resulting in a strong increase in tagatose productivity (yield of 9.1 g/Lh) and a moderate increment in fructose productivity.

Tri-enzymatic co-immobilized derivatives could be more efficient biocatalysts to minimize or to avoid product inhibition during mono-reactor operation, but diverse conditions must be analyzed in detail with relation to coupling, stabilization, and operation mode. These studies are the aims of a forthcoming paper. 

## Figures and Tables

**Table 1:** Immobilization and stabilization of l-arabinose isomerase onto Eupergit C 250 L and Eupergit C ^1^.

Support	Alkaline Incubation at pH 8.5 (h)	Applied Protein (mg/g of gel)	Protein Imm. Yield (%)	Imm. Activity Yield (%)	Half Life (h)
Eupergit C 250 L	0	4.1 ± 0.1	100 ± 1	89 ± 3	65 ± 6
	7	----------	-----------	81 ± 3	214 ± 6
	24	-----------	-----------	73 ± 6	308 ± 2
Eupergit C	0	4.1 ± 0.1	100 ± 1	71 ± 6	95 ± 3
	7	----------	-----------	59 ± 3	330 ± 8
	24	----------	----------	51 ± 3	379 ± 2

^1^ Immobilization was carried out by incubating 1.6 U of l-arabinose isomerase preparation (0.40 U/mg) with 1 g of corresponding support in 1 M potassium phosphate buffer pH 7.5 for 24 h. Blocking was then performed with glycine 3 M (indicated as 0 h in the table) or incubation was carried out at pH 8.5 for 7 or 24 h followed by blocking with glycine. Kinetics of inactivation in batch at 50 °C for derivatives was monitored for 1–55 h. Half-life of native enzymes (in equivalent amount to derivatives) was 6 h under these conditions. Eupergit C and Eupergit C 250 L unblocked derivatives exhibited half-lives of 4 h and 7 h, respectively. Results are the means of triplicate determinations ± SD.

**Table 2:** Immobilization of d-xylose (d-glucose) isomerase onto Eupergit C and Eupergit C 250 L and stabilization of the derivatives by glycine blocking or alkaline treatment. ^1^

Support	Alkaline Incubation at pH 8.5 (h)	Applied Protein (mg/g of gel)	Protein Imm. Yield (%)	Imm. Activity Yield (%)	Half Life (h)
Eupergit C 250 L	0	1.9 ± 0.2	100 ± 1	98 ± 1	210 ± 6
	7	----------	-----------	91 ± 1	360 ± 6
	24	-----------	-----------	84 ± 1	491 ± 2
Eupergit C	0	1.9 ± 0.2	100 ± 1	91 ± 1	230 ± 2
	7	----------	-----------	79 ± 1	389 ± 6
	24	----------	----------	66 ± 1	554 ± 6

^1^ Immobilization of d-xylose (d-glucose) isomerase was carried out by incubating 5.8 U of enzyme preparation (3 U/mg) with 1 g of corresponding support in 1 M potassium phosphate buffer pH 7.5 for 24 h. Blocking was then performed with 3 M glycine (indicated as 0 h in table) or derivatives were incubated at pH 8.5 for 7 or 24 h and then blocked with glycine. Kinetics of batchwise inactivation at 50 °C for derivatives was monitored for 1–55 h. Half-life of native enzyme (equivalent amount) was 23 h under these conditions. Eupergit C and Eupergit C 250 L unblocked derivatives exhibited half-lives of 19 h and 24 h, respectively. Results are means of triplicate determinations ± SD.

**Table 3:** Kinetic parameters of soluble l-arabinose (d-galactose) isomerase and derivatives. ^1^

Biocatalyst	Temperature (°C)	K_m_ (mM)	V_max_ (nM/min)
Native enzyme	30	100 ± 7	10 ± 3
	40	70 ± 2	40 ± 3
	50	34 ± 2	80 ± 6
Eupergit C derivative	30	170 ± 2	1 ± 0.7
	40	120 ± 2	35 ± 1
	50	50 ± 2	60 ± 6
Eupergit C 250 L derivative	30	127 ± 6	3 ± 0.1
	40	100 ± 2	45 ± 1
	50	40 ± 2	70 ± 2

^1^ Results are means of triplicate determinations ± SD.

**Table 4:** Kinetic parameters for soluble d-xylose (d-glucose) isomerase and derivatives. ^1^

Biocatalyst	Temperature (°C)	K_m_ (mM)	V_max_ (nM/min)
Native enzyme	30	365 ± 3	49 ± 8
	40	315 ± 2	68 ± 4
	50	265 ± 2	176 ± 2
Eupergit C derivative	30	925 ± 3	17 ± 2
	40	802 ± 2	59 ± 5
	50	750 ± 2	159 ± 2
Eupergit C 250 L derivative	30	905 ± 3	31 ± 2
	40	775 ± 6	65 ± 5
	50	672 ± 7	161 ± 6

^1^ Results are means of triplicate determinations ± SD.

**Table 5:** Lactolysis and isomerization in Mozzarella cheese whey at 50 °C by tri-enzymatic systems ^1^.

System	Lactolysis (%)	Tagatose (%) ^2^	Fructose (%) ^2^
Soluble enzymes	76 ± 1	22 ± 3	21 ± 1
Immobilized derivatives (sequential use)	86 ± 1	31 ± 2	24 ± 2
Immobilized derivatives (simultaneous use)	93 ± 3	40 ± 1	29 ± 3

^1^ Results are means of triplicate determinations ± SD; ^2^ Conversion percentages (6 h operation) according to HPLC analysis (see supporting information in supplementary materials).

## Supplementary Materials

Supplementary materials are available online.
