# Supplementary material for: Immobilized Trienzymatic System with Enhanced Stabilization for the Biotransformation of Lactose
Source: Molecules. 2017 Feb 22;22(2):284. doi: 10.3390/molecules22020284 (PMC6155631; doi:10.3390/molecules22020284)
Supplement: Supplementary file 1 [file molecules-22-00284-s001.pdf]

Supplementary Materials: Immobilized Trienzymatic System with Enhanced Stabilization for the Biotransformation of Lactose

Pedro Torres and Francisco Batista-Viera

Table S1. Kinetics of process for biotransformation of lactose in Mozzarella cheese whey at 50 °C using immobilized tri-enzymatic system.

System	Time (h)	Tagatose (%) [1]	Fructose (%) [1]
Soluble enzymes	0	0	0
	1	7	8
	2	10	10
	3	13	13
	4	16	17
	5	19	19
	6	22	21
Immobilized derivatives (sequential use)	0	0	0
	1	10	1
	2	16	7
	3	19	10
	4	21	13
	5	27	19
	6	31	24
Immobilized derivatives (simultaneous use)	0	0	0
	1	11	5
	2	17	8
	3	20	11
	4	25	17
	5	33	24
	6	40	29

[1] Conversion percentage (D-galactose to D-tagatose and D-glucose to D-fructose).

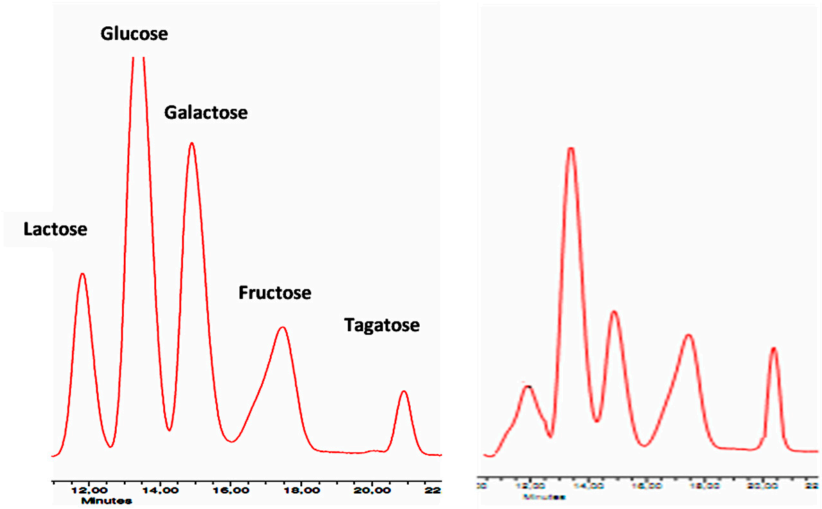

(**a**) (**b**)

Figure S1. HPLC profile of: (**a**) Sugar standards; (**b**) Products from simultaneuos lactolysis, glucose and galactose isomerization in Mozzarella cheese whey with the trienzymatic system (single enzyme derivative). Reaction time: 6 h.
